# Quantification of Myocardial Biomarkers in Sudden Cardiac Deaths Using a Rapid Immunofluorescence Method for Simultaneous Biomarker Analysis

**DOI:** 10.3390/biomedicines13010193

**Published:** 2025-01-14

**Authors:** Matteo Antonio Sacco, Valerio Riccardo Aquila, Saverio Gualtieri, Roberto Raffaele, Maria Cristina Verrina, Lucia Tarda, Santo Gratteri, Isabella Aquila

**Affiliations:** 1 Institute of Legal Medicine, Department of Medical and Surgical Sciences, “Magna Graecia” University, 88100 Catanzaro, Italy; matteoantoniosacco@gmail.com (M.A.S.); saverio.gualtieri@studenti.unicz.it (S.G.); raro81@libero.it (R.R.); mariacristina.verrina@studenti.unicz.it (M.C.V.); lucia.tarda@studenti.unicz.it (L.T.); gratteri@unicz.it (S.G.); 2 Department of Medical and Surgical Sciences, “Magna Graecia” University, 88100 Catanzaro, Italy; valerioriccardo.aquila@studenti.unicz.it

**Keywords:** sudden cardiac deaths, cardiac markers, troponins, forensic sciences

## Abstract

Background/Objectives: Differential diagnosis of sudden cardiac death (SCD) remains challenging, particularly in cases lacking evident structural abnormalities. Cardiac markers have been proposed as useful tools for this differentiation in forensic contexts. However, key issues include the influence of postmortem interval (PMI) on marker stability and the limitations of traditional approaches that focus on pericardial fluid, which requires invasive sampling compared to peripheral blood. This study aimed to evaluate the potential of cardiac markers in peripheral blood for diagnosing SCD, addressing methodological concerns related to PMI, hemolysis, and sample handling. Methods: This study analyzed 5 cardiac markers (creatine kinase-MB [CK-MB], myoglobin, troponin I [TnI], BNP, and D-dimer) in peripheral blood samples from 42 autopsied cadavers, divided into an SCD group and a control group. Marker levels were quantified using immunofluorescence, with cases meticulously selected to exclude confounding factors such as chronic diseases, pulmonary thromboembolism, and drowning. The study also accounted for potential degradation due to PMI, and evaluated the accuracy of point-of-care testing (POCT) in forensic samples. Results: The study identified statistically significant differences in myoglobin and TnI levels between the SCD group and the control group, though myoglobin’s diagnostic reliability remains limited due to its lack of specificity for myocardial injury. TnI emerged as a more robust marker for SCD. Contrary to prior concerns, PMI showed no significant correlation with marker levels in samples handled without freeze–thaw cycles. Issues related to hemolysis were addressed, and no significant effects were observed from resuscitation maneuvers. Conclusions: This study supports the potential use of cardiac markers, particularly TnI, in peripheral blood for postmortem SCD diagnosis, emphasizing the importance of rapid and systematic analysis to minimize hemolysis-related variability. While further validation is needed to confirm these findings, this approach offers a less invasive, economical, and practical method for forensic investigations.

## 1. Introduction

**Figure 1:**
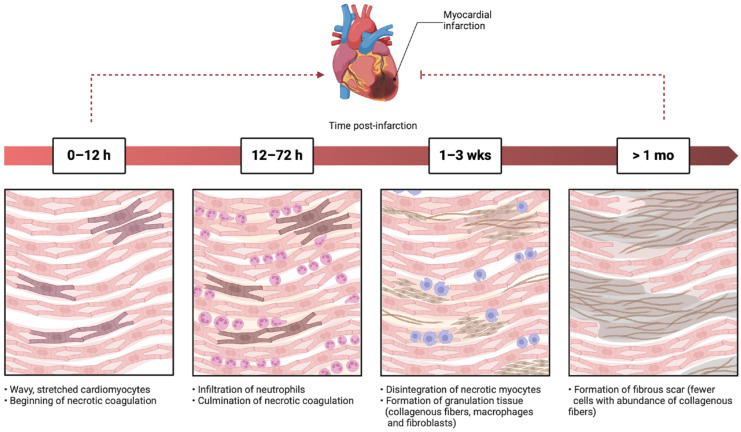
Evolution of myocardial infarction by microscopic analysis (adapted with Biorender.com).

Sudden cardiac deaths (SCDs) diagnostics are a major challenge for the forensic pathologist [1]. To date, the literature estimates that a notable number of SCDs do not present conclusive signs at autopsy, or only show findings indicative of cardiac death in the absence of pathognomonic evidence [2]. Among these, there is hypostasis distributed on the face and neck, and plurivisceral congestion. Also, from a microscopic point of view, histopathological examination of the heart can have limitations [3]. In particular, in cases of acute myocardial ischemia, it is likely that there are no findings of myocardial necrosis or inflammatory infiltrates that can be detected microscopically, by virtue of the timing related to the evolution of the myocardial damage (Figure 1).

In these cases, it is therefore necessary to carefully examine the integrity of the coronary arteries and evaluate the degree of stenosis of the lumen. Immunohistochemistry can offer a contribution in these cases [4]. Furthermore, it is necessary to investigate anamnestic and clinical data including the collection of prodromal signs or symptoms in the hours preceding the event. The lack of pathognomonic evidence at the autopsy examination can have an impact in the judicial field, especially in cases where it is necessary to reconstruct the cause of death with certainty and demonstrate any profiles of responsibility in the death.

In the clinic, cardiac markers exhibit specific patterns of elevation that can be used to determine the timing of myocardial injury. However, their importance in forensic autopsies has gained attention, as they can provide information regarding the cardiac status at the time of death. The utility of cardiac markers in postmortem investigations is still being explored. In fact, even today there is no univocal consensus on the diagnostic value of many cardiac markers. The majority of papers have focused on the analysis of pericardial fluid, whose sampling requires an invasive approach to the cadaver [5,6]. Few works have analyzed alternative biological matrices on cadavers, such as peripheral blood, with varied results [6,7]. Furthermore, some works have reported the risk of troponins varying with PMI or resuscitation maneuvers [8,9]. In this work, we quantified the levels of five different cardiac biomarkers in peripheral blood in two groups, classified as group 1 (SCDs) and group 2 (controls). The aim of the work is to evaluate the potential application of the measurement of cardiac marker levels in blood as an aid for the forensic diagnosis of cardiac deaths. Variables such as PMI and resuscitation maneuvers were considered in the analysis.

## 2. Materials and Methods

### 2.1. Analyzed Cases

The work obtained the approval of the Calabria Region Ethics Committee, with protocol no. 69 of 26 February 2024. We examined a series of autopsies carried out at the Institute of Legal Medicine, Department of Medical and Surgical Sciences of the Magna Graecia University of Catanzaro (n = 42; PMI between 41 and 194 h, with a median of 87.0 h). Of these, 30 were males and 12 were females with an age between 0 and 89 (median equal to 56.5). The case series was then divided into two groups on the basis of the autopsy and histopathological and toxicological findings. Group I (SCDs) included cases that fell within the definition of SCD, showed signs attributable to myocardial ischemia or acute myocardial infarction, or were otherwise concluded with this diagnosis of death [9]. Group II (controls) included cases of subjects who died from proven violent causes of non-cardiac origin, i.e., hanging, gunshot wounds, falling from height, or other causes.

For each subject included, data relating to sex, age, cause of death, and elapsed PMI, understood as the time between the observation of death and the time of the autopsy with analysis of the samples, were collected. The dates and times of death were acquired through analysis of the death reports in the case of medical intervention, as well as from the cadaveric findings carried out in the case of a judicial inspection.

### 2.2. Autopsy and Post-Autopsy Investigations

The heart was examined at autopsy with gross analysis, opening of the cardiac chambers, and visualization of the coronary arteries. Subsequently, the heart was completely fixed in formalin. The visualization of the heart after fixation was then repeated, and the preparations for histopathological investigations were prepared [10]. The resulting slides were then viewed under the microscope. Toxicological analyses were carried out in all cases by screening urine and peripheral blood.

### 2.3. Sample Collection

Peripheral blood was collected using a sterile syringe after puncture of the subclavian vein, following a standardized protocol designed to minimize contamination and hemolysis. The sample was immediately transferred into a sterile tube, ensuring minimal handling to avoid additional hemolysis. A volume of 1 mL was promptly diluted with physiological solution in a 1:4 ratio to prevent postmortem coagulation phenomena, ensuring the sample’s consistency for analysis. The protocol also required that the analysis be conducted immediately after sample collection, without freezing, to further reduce the risk of hemolysis and degradation of biomarkers. The sample was gently mixed four times [11] to homogenize the dilution without causing mechanical hemolysis. Five drops of the sample were extracted (volume equal to 0.2 mL) and transferred with a special pipette onto the disposable “Triage Profiler SOB Panel” device—Quidel (San Diego, CA, USA). The device allowed us to simultaneously measure the levels of all the chosen markers, i.e., CK-MB, myoglobin, troponin I, BNP, and D-dimer in the timing. The device featured a well inside the test cassette with an absorbent filter. From the well, the sample migrated through a strip containing antibodies against the sought antigens, i.e., myocardial markers. Therefore, if present, the antigens bound to the specific antibody labeled with europium. At the end of the incubation, which lasted approximately ten minutes, visible macroscopically as the end of the migration of the sample on the strip, the device was ready for reading.

### 2.4. Analytical Phase

**Figure 2:**
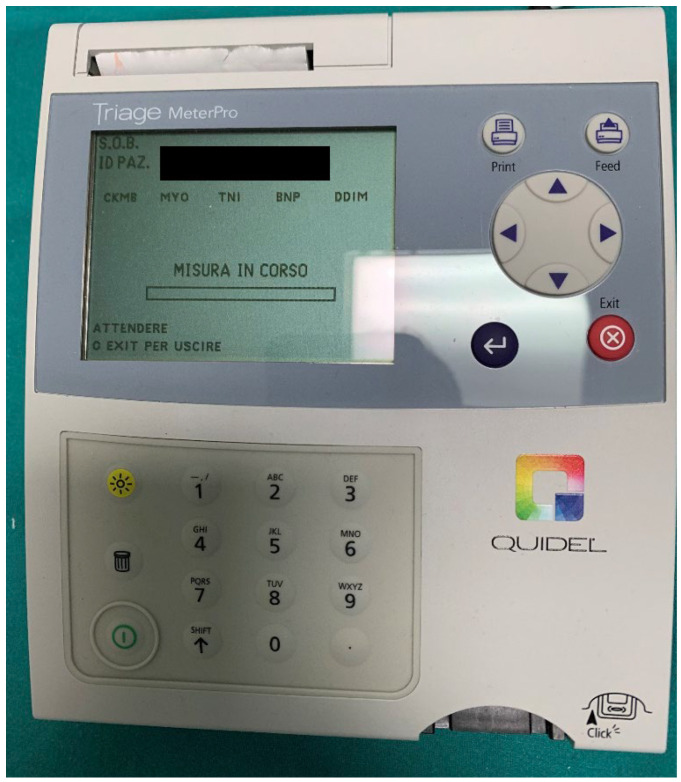
Detection equipment used for the study.

The detection equipment used in this study is commercially available and marketed as the SOFIA FIA (Fluorescent Immunoassay Analyzer) by Quidel Corporation (San Diego, CA, USA). This portable device is designed for rapid analysis of specific biomarkers and is equipped with UV fluorescence detection (Quidel Corporation, San Diego, CA, USA) integrated within the machine. The device targets the five selected markers (CK-MB, myoglobin, troponin I, BNP, and D-dimer) as part of a pre-configured assay panel optimized for clinical and forensic applications. Weighing 1.36 kg, the device is easily transportable, and capable of operating with an electronic socket or independently on batteries, making it suitable for both laboratory and field settings (Figure 2).

The device utilizes europium-labeled antigen–antibody fluorescence detection, which provides high sensitivity and specificity for biomarker quantification. Before each analysis, a pre-test calibration phase was performed to ensure the accuracy and reliability of the device. This calibration step was automated and took approximately one minute. Following calibration, the subject’s personal data were entered, and the diluted sample was introduced into the device.

Under UV irradiation, the europium-labeled antigen–antibody sandwich complex produced fluorescence, with intensity directly proportional to the concentration of the target protein in the original sample. The detection relied on mouse and goat mono- and polyclonal antibodies, ensuring high specificity for the chosen biomarkers (CK-MB, myoglobin, troponin I, BNP, and D-dimer). The fluorescence signal was compared to a pre-established reference cutoff to determine the concentration levels of each marker.

**Figure 3:**
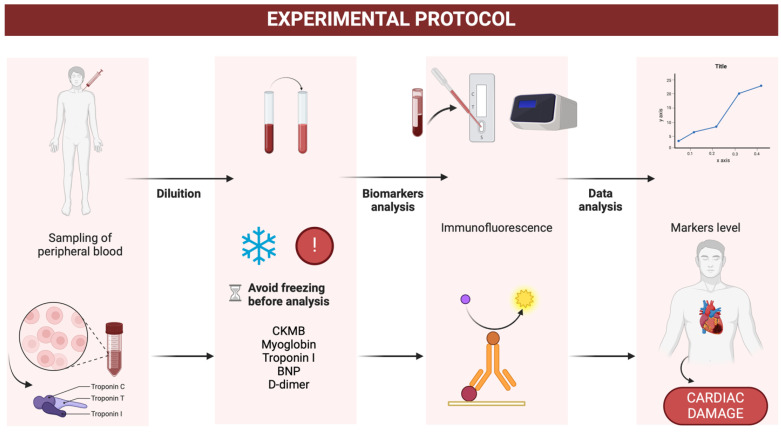
Experimental analysis protocol on postmortem blood (created with Biorender.com).

Each analysis was completed within 10 min, offering a rapid and efficient workflow. To ensure reproducibility and minimize errors, the reading of each sample was conducted in duplicate, and the mean values of the biomarker levels were calculated. This robust methodology ensured the reliability of the results, making the system suitable for the fast-paced requirements of forensic investigations. (Figure 3) (Table 1).

The described method utilizes monoclonal and polyclonal antibodies that have been rigorously validated for their specificity and ability to accurately detect key biomarkers including CK-MB, myoglobin, troponin I, BNP, and D-dimer. These antibodies have been tailored to recognize unique epitopes specific to each biomarker, ensuring minimal cross-reactivity with other proteins. For instance, according to the manufacturer, the troponin I antibodies are specifically designed to detect the cardiac isoform of troponin I, with no significant interference from skeletal muscle isoforms. The specificity of troponin I detection remains at 100% within the first 6 h post sample collection, and slightly decreases to 90.1% after 24 h. This high specificity makes troponin I an excellent marker for cardiac injury.

The specificity of CK-MB and myoglobin antibodies also shows strong performance, with values ranging from 82.2% to 91% for CK-MB, and 67.8% to 81.8% for myoglobin. These antibodies are particularly sensitive to myocardial tissue, although myoglobin detection may be influenced by skeletal muscle injuries. For BNP, the antibodies are directed towards circulating fragments released during cardiac stress. The specificity for BNP is well maintained throughout a wide range of concentrations, ensuring accurate detection even in the presence of other similar peptides. D-dimer antibodies also demonstrate specificity by targeting fibrin degradation products, with minimal interference from other coagulation factors, providing valuable information in thrombotic conditions. According to the manufacturer, the device has shown robust performance even in challenging sample conditions, such as those affected by hemolysis, lipemia, or icterus.

### 2.5. Statistical Investigations

The study included peripheral blood samples taken from subjects in two groups: an SCD group and a control group (Supplementary Materials). Variables in the dataset included specific demographics and biomarkers. The BNP variable was excluded from the analysis due to low variability. The biomarkers analyzed were CKMB, MYO, TnI and D-dimer. Categorical variables such as sex, group, cause of death and maneuver were converted into factors. Variable labels have been standardized for clarity. The data were preliminarily explored using summary functions and graphical visualizations. Boxplots were generated for each biomarker to examine the distribution between the two groups. Differences between groups for each biomarker were analyzed using linear regression models and Wilcoxon tests if the assumptions of normality and homoscedasticity were not met. Links between age and biomarker levels were graphically explored using scatter plots with regression lines.

The relationship between PMI, maneuver, and the biomarkers MYO and TnI was investigated using linear models that included the interaction between group and PMI or group and maneuver. The maneuver was coded as a dummy variable (−1 and 1) to directly carry out zero mean contrasts. Finally, it was tested whether the addition of PMI and maneuver improved the fit of the data compared to models that only considered the group; this was made possible through the use of ANOVA in the model comparison, since we tested nested models.

Statistical analyses and visualizations were performed using R software version 4.3.0, with the ggplot2, effects, cowplot, and effectsize libraries [12,13,14,15,16].

The ROC curve analysis was performed to evaluate the effectiveness of two biomarkers, myoglobin (MYO) and troponin I (TnI), in predicting the “Experimental” condition versus the “Control” group. For each biomarker, a logistic regression model was built using the glm() function with the binary response variable “Group” and the biomarker as a predictor. The linearity of the logit was checked, and deviance residuals were examined for outliers or influential cases. Predicted probabilities were then computed using the predict() function with the “response” type of output. ROC curves were generated for each model, and the optimal cutoff values were determined based on the maximum combined sensitivity and specificity. A combined model, including both biomarkers, was also developed to compare the diagnostic performance of the individual biomarkers. The resulting confusion matrices for each model were then analyzed to evaluate the performance in terms of true positives (TPs), false positives (FPs), true negatives (TNs), and false negatives (FNs) (Table 2 and Table 3).

## 3. Results

Using regression models, no statistically significant differences were found between the groups for D-dimer (t = 0.08; *p*-value = 0.94). In the case of CKMB, we used a Wilcoxon rank sum test, which did not detect statistically significant differences between the distributions of the two groups (W = 244; *p*-value = 0.55).

**Figure 4:**
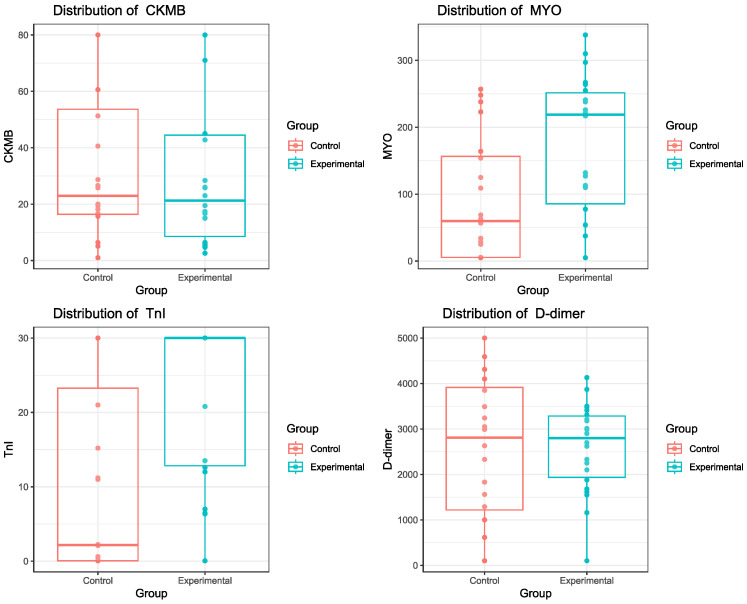
Statistical distribution of markers in the two groups.

Mean levels of MYO were statistically higher in the experimental group (t = 2.72; *p*-value = 0.009). Since we found an effect, we calculated the appropriate effect size, which in this case was a Cohen’s d = 0.84, indicating a large effect. As we can see from the boxplot, the experimental group is divided into two halves. The upper half is characterized by two Arit, five Inf, one Ins, and four Isc, while the bottom half included one Arit, one Diss, two Ins, and six Isc. We also tested if the PMI was related to an increase in myoglobin concentration. We estimated another regression model including PMI centered on its mean and hypothesized an interaction between PMI and group. However, we did not find any effect of PMI, and the additional predictor did not explain the significantly higher variance in the model. Indeed, comparing this model with the one including only the group as a predictor using a nested ANOVA returned a non-significant *p*-value (F = 0.7; *p*-value = 0.5). Additionally, we tested the use of cardiac maneuvers as a predictor but found no effect. The comparison between this model and the one with only the group as a predictor using ANOVA showed no improvement in explained variability (F = 0.29; *p*-value = 0.75). Finally, we added age as a predictor in the model for myoglobin concentration. This model revealed a significant interaction between group and age (F = 3.3753; *p*-value = 0.045), suggesting that the effect of age on myoglobin levels may vary within the group. Moreover, we still observed a significant difference between the experimental and control groups in the model with age (t = 2.72, *p*-value = 0.009). To investigate differences in TnI concentrations between the two groups, we first estimated a regression model, but due to the ceiling effect, we violated the normality of residuals assumption. We proceeded to compare the two distributions using a Wilcoxon rank sum test, which provided a statistically significant *p*-value (W = 106; *p*-value = 0.002). We found higher levels of TnI in the experimental group and calculated the rank biserial correlation between variables (rbis = 0.47), indicating a moderate effect. We also tested if the addition of PMI and maneuvers, respectively, explained a statistically higher variance in the model. In this case, ANOVA showed no improvement both in PMI (F = 0.62; *p*-value = 0.54) and maneuvers (F = 0.58; *p*-value = 0.56) (Figure 3 and Figure 4). We also included age in the model for TnI, but no significant effect was found for age or its interaction with group (F = 1.14; *p*-value = 0.33) (Figure 4).

**Figure 5:**
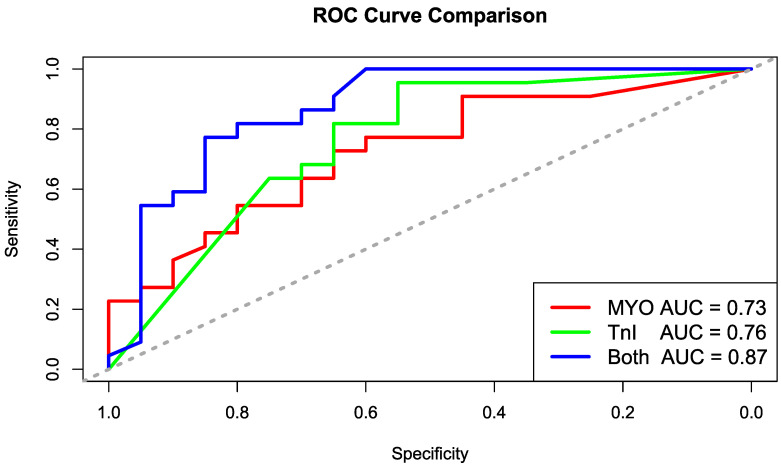
ROC curve comparison.

For the MYO biomarker, the logistic regression model showed an area under the curve (AUC) of 0.73. The optimal cutoff for MYO, determined based on the maximum sensitivity and specificity, was found to be 130. At this cutoff, the sensitivity was 0.73 and the specificity was 0.65. For TnI, the AUC was 0.76, with a cutoff of 21. At the optimal cutoff for TnI, the sensitivity was 0.82 and the specificity was 0.65. A combined model, including both biomarkers (MYO and TnI), produced a higher AUC of 0.87, suggesting improved classification. The sensitivity for the combined model was 0.77, and the specificity was 0.85. The ROC curves for the individual biomarkers and the combined model were plotted (Figure 5), and statistical tests comparing the ROC curves showed no significant difference between MYO and TnI (*p* = 0.775), while the comparison between MYO and the combined model revealed a significant difference (*p* = 0.046). The comparison between TnI and the combined model showed no significant difference (*p* = 0.091). After correcting for multiple comparisons using the Bonferroni method, no *p*-values were found to be statistically significant (Table 4, Table 5 and Table 6).

## 4. Discussion

The scientific literature has suggested the possibility of using markers of myocardial damage for postmortem diagnostics in SCD through various experimental models, although to date, there is no uniform consensus on the most useful biological matrix and results [17,18,19,20]. In our study, we evaluated a series of 42 subjects subjected to autopsy, using a multiparametric analytical strategy with immunofluorescence. Peripheral blood was chosen as the biological matrix for its ease of collection from cadavers, despite being less studied in comparison to other matrices like pericardial fluid. The choice of the five biomarkers was instead aimed at markers commonly applied in the clinic in the triage of chest pain and dyspnea, as they are highly sensitive to ischemic myocardial damage and cardiac structural remodeling.

Among the biomarkers analyzed, troponin I, along with troponin T and troponin C, forms part of the troponin complex that regulates cardiac muscle contraction. Upon myocardial injury, cytosolic troponin is released initially, followed by troponin from the sarcomeres as the damage progresses [21,22,23,24,25,26,27,28,29]. In our study, troponin I (TnI) demonstrated significantly higher levels in the SCD group compared to controls. This aligns with its well-known specificity for myocardial injury and minimal expression in non-cardiac tissues, such as skeletal muscle. The specificity and stability of TnI under controlled conditions, such as immediate postmortem analysis without freeze–thaw cycles, reinforce its potential for postmortem diagnostics. Previous studies (e.g., Cina et al., 1998; Pérez-Cárceles et al., 2004) also support the utility of TnI in postmortem settings [27,30]. However, other reports, such as those by Tomásková et al. (2010), have questioned its diagnostic efficacy [7], likely due to variations in postmortem handling protocols, sample degradation, or differences in biological matrices. For instance, recent findings have suggested that these markers may lack specificity or stability in postmortem settings, due to factors such as hemolysis, autolysis, and variability in postmortem intervals [31].

Despite these challenges, our results demonstrate that TnI is a robust marker for distinguishing SCD cases, aligning with recent reviews [32,33,34].

**Figure 6:**
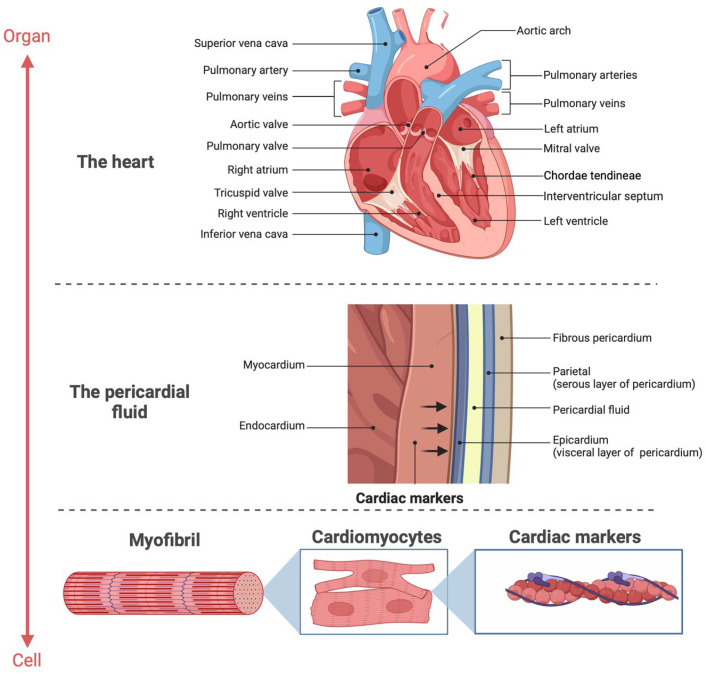
Release of markers of cardiac damage into the pericardial fluid (created with Biorender.com).

Conversely, CK-MB did not exhibit significant differences between the SCD and control groups, confirming its limited utility in postmortem settings due to its susceptibility to hemolysis, autolysis, and proteolysis [24]. The molecular structure of CK-MB, a dimeric protein, likely contributes to its instability in peripheral blood, whereas its concentration is better preserved in pericardial fluid, a more protected matrix (Figure 6). Similar limitations apply to BNP, whose postmortem instability renders it unsuitable for diagnostics, as well as D-dimer, which is non-specific and influenced by numerous conditions shared between SCD and control groups [34,35].

Marker stability is a critical factor for the diagnostic utility of TnI in postmortem settings. As highlighted in prior studies [36], TnI demonstrates relatively good stability under controlled conditions, particularly when blood samples are processed promptly after autopsy and without freeze–thaw cycles. In our study, the stability of TnI was supported by consistent results across a wide range of postmortem intervals (PMI: 41–194 h). This highlights the potential of TnI for reliable postmortem diagnostics, provided that stringent sample handling protocols are followed. Future work could extend this research by systematically testing TnI stability across different biological matrices (e.g., pericardial fluid) and under varying environmental conditions to further validate its robustness.

In addition to conventional markers like TnI, novel cardiac markers have been proposed for postmortem applications. For instance, quantitative mRNA measurement of atrial and brain natriuretic peptides (ANP and BNP) and endoplasmic reticulum stress-related secretory proteins, which have been investigated as promising biomarkers for cardiac-specific injuries in both clinical and forensic settings, demonstrate high specificity and stability in controlled studies [36,37]. These markers may provide complementary insights when traditional markers are less reliable, due to factors like hemolysis or extended PMI. Incorporating these non-canonical markers into multiparametric diagnostic strategies could enhance the sensitivity and specificity of postmortem analyses. Further studies are warranted to explore their applicability and stability in diverse forensic contexts.

Among the limitations, the study’s small sample size (n = 22 for SCD cases) and the heterogeneity of SCD cause the introduction of variability that complicates result interpretation. The subgroup analysis of biomarker levels showed no statistically significant differences between patients with myocardial ischemia/infarction and those with aortic dissection, arrhythmias, or heart failure. Although both myoglobin and troponin I were elevated in all SCD cases, the small sample sizes, particularly in the aortic dissection (n = 1) and arrhythmia (n = 3) groups, limit the interpretability of these findings. This highlights the need for larger studies with more homogenous subgroup distributions to confirm whether certain biomarkers may differentially indicate the specific causes of SCD. Additionally, future research could benefit from exploring complementary markers or integrating clinical and histopathological data to enhance subgroup discrimination.

Additionally, cases of SCD attributed to cardiac arrhythmias, often categorized as sudden unexplained deaths with negative autopsy findings, present unique challenges. In these cases, myocardial damage may be absent, with electrophysiological disturbances as the primary mechanism. Consequently, markers like TnI and myoglobin, which rely on myocardial damage for elevation, may remain within normal ranges. This underscores the need for complementary diagnostic approaches, such as genetic testing for channelopathies or advanced electrophysiological studies.

Finally, although postmortem intervals (PMI) ranged from 41 to 194 h, samples were analyzed immediately after autopsy without refrigeration–thawing cycles, minimizing hemolysis. While this approach enhanced marker stability, future studies should explore high-sensitivity techniques such as mass spectrometry to complement POCT analyses, particularly for hemolysis-prone markers [38,39,40,41,42,43,44,45,46,47,48,49,50,51,52,53]. Finally, rigor mortis, as a physiological process following death, could contribute to variations in biomarker levels independently of postmortem interval (PMI). While PMI was included in our analysis and shown not to significantly influence biomarker levels, the exact time of the event (i.e., the time of death) might provide a more accurate parameter for assessing the dynamic changes in these biomarkers. However, due to the retrospective nature of this study, precise information regarding the time of the event was not consistently available. Cases with chronic conditions known to significantly influence cardiac biomarkers, such as chronic kidney disease or cerebrovascular stroke, were excluded to minimize confounding. Despite these exclusions, we acknowledge that residual confounding factors may still exist due to the heterogeneity of non-cardiac causes of death. Future studies with a prospective design and detailed documentation of the time of death will be crucial to disentangling the specific effects of rigor mortis and other perimortem processes on cardiac biomarkers. Integrating data from multiple biomarkers using advanced statistical methods or artificial intelligence could enhance diagnostic accuracy by identifying complex patterns and interactions. In conclusion, the work opens new research perspectives on the application of cardiac marker dosing in the postmortem diagnostics of SCD through innovative and rapid methods. Our study highlights the potential for using TnI as a reliable marker for postmortem diagnostics in SCD, particularly when sample handling is optimized. Further research integrating multiple biomarkers and advanced analytical methods will help refine these diagnostic tools and overcome the current limitations.

## Figures and Tables

**Table 1:** Analytical performance parameters and technical details of the rapid test system.

Parameter	Details
Device Name	SOFIA FIA (Fluorescent Immunoassay Analyzer)
Manufacturer	Quidel (San Diego, USA)
Portability	Lightweight (1.36 kg), operates with electronic socket or batteries
Markers Measured	CK-MB, myoglobin, troponin I (TnI), BNP, and D-dimer
Detection Principle	Europium-labeled antigen–antibody fluorescence sandwich assay
Sample Requirements	0.2 mL of peripheral blood diluted in a 1:4 ratio with physiological solution
Calibration	Pre-test calibration performed for each run to ensure accuracy
Analysis Time	Approximately 10 min per sample
Sample Handling	Immediate analysis after collection to minimize hemolysis and degradation
Fluorescence Reader	UV-induced fluorescence detection proportional to protein concentration
Accuracy	Duplicate readings were performed; mean values were used for final results
Technical Performance	Comparison to a reference cutoff using mouse and goat mono- and polyclonal antibodies

**Table 2:** Group I data (SCDs).

Case	Sex	Age (Years)	PMI (h)	Cause of Death	Primary Cause of Death (Pathology)	CK-MB (ng/mL)	MYO (ng/mL)	TnI (ng/mL)	BNP (pg/mL)	D-Dimer (ng/mL)	Resuscitative Maneuvers
1	F	79	167	Acute myocardial ischemia	Coronary artery atherosclerosis with 80% luminal stenosis	80.0	5.0	30.0	5.0	2100	Yes
2	M	74	158	Acute myocardial ischemia	Coronary artery atherosclerosis with 75% luminal stenosis	80.0	110	30.0	5.0	3500	Yes
3	M	83	94	Acute myocardial ischemia	Coronary artery atherosclerosis with plaque rupture	71.0	54	30.0	5.0	2900	No
4	M	71	95	Acute myocardial infarction	Coronary thrombosis with 85% atherosclerotic stenosis	5.7	241	6.35	5.0	2250	Yes
5	M	49	44	Acute heart failure	Left ventricular hypertrophy with decompensated heart failure	17.4	264	7.0	5.0	1550	Yes
6	F	55	180	Arrhythmia	Long QT syndrome with documented ventricular arrhythmias	25.8	338	0.05	5.0	100	No
7	M	52	99	Acute myocardial ischemia	Coronary artery atherosclerosis with 90% stenosis	45	132	30.0	5.0	3870	Yes
8	F	81	72	Acute myocardial ischemia	Severe multivessel coronary artery disease with 85% stenosis	80	5.0	30.0	5.0	1880	Yes
9	M	54	127	Acute myocardial infarction	Acute coronary thrombosis and rupture of a vulnerable plaque	2.6	221	6.46	5.0	1160	Yes
10	M	73	89	Acute myocardial ischemia	Coronary artery atherosclerosis with 70% luminal stenosis	5.2	238	30.0	5.0	3210	No
11	M	68	131	Acute myocardial ischemia	Coronary artery atherosclerosis with 85% stenosis	28.4	310	12.6	5.0	1620	Yes
12	F	89	50	Acute heart failure	Dilated cardiomyopathy with reduced ejection fraction	6.2	217	30.0	5.0	2700	No
13	M	45	88	Acute myocardial infarction	Acute coronary thrombosis with STEMI findings	4.7	226	30.0	5.0	3410	No
14	M	68	194	Acute myocardial infarction	Coronary artery disease with diffuse calcified plaques	16.7	297	12.0	5.0	1680	Yes
15	M	62	109	Acute myocardial infarction	Coronary thrombosis with 80% atherosclerotic stenosis	19.5	255	13.5	5.0	3010	Yes
16	M	60	116	Aortic dissection	Acute aortic dissection (Stanford Type A) with rupture	15.1	113	30.0	5.0	2990	Yes
17	M	58	127	Acute myocardial ischemia	Coronary artery atherosclerosis with 60% stenosis and plaque erosion	80.0	53.9	30.0	5.0	3480	No
18	M	71	70	Arrhythmia	Long QT syndrome with documented ventricular arrhythmias	42.8	77.5	30.0	5.0	2330	No
19	F	22	49	Arrhythmia	Hypertrophic cardiomyopathy with arrhythmic sudden death	15.0	241	20.8	5.0	3180	Yes
20	M	50	75	Acute myocardial infarction	Coronary thrombosis with STEMI findings	6.4	267	30.0	5.0	4130	No
21	F	70	71	Acute heart failure	Severe pulmonary hypertension with acute decompensation	23.0	37.6	30.0	11.6	2620	Yes
22	M	79	117	Acute heart failure	Dilated cardiomyopathy with secondary heart failure	26.0	127.0	30.0	5.0	3310	Yes

**Table 3:** Group II data (controls).

Case	Sex	Age (Years)	PMI (h)	Cause of Death	CK-MB (ng/mL)	MYO (ng/mL)	TnI (ng/mL)	BNP (pg/mL)	D-Dimer (ng/mL)	Resuscitative Maneuvers
1	M	69	107	Head injury	80.0	5.0	0.05	38.0	1560	No
2	F	35	41	Hanging	18.2	5.0	0.05	5.0	100	No
3	M	39	68	Gunshot injuries	26.6	5.0	0.05	5.0	100	No
4	M	76	55	Fire injuries	1.0	154	15.2	5.5	4310	No
5	F	45	51	Falling from height	15.8	238	30	5.0	1830	No
6	F	60	65	Slaughtering	80	5.0	30	100	1000	No
7	F	24	152	Hemorrhagic shock	15.7	34	0.05	5.0	1290	Yes
8	M	25	69	Gunshot injuries	20.0	62.8	11.2	5.0	5000	No
9	F	31	69	Gunshot injuries	28.7	164	0.20	5.0	5000	No
10	M	36	45	Falling from height	25.8	5.0	0.05	5.0	100	No
11	M	50	54	Falling from height	40.6	125	21.0	5.0	4100	No
12	M	61	54	Road traffic accident polytrauma	60.6	68.9	30.0	5.0	3850	No
13	M	40	61	Falling from height	19.8	257	11.0	5.0	3050	No
14	F	72	43	Hanging	19.4	248	2.22	5.0	2990	No
15	M	58	127	Strangulation	16.6	223	2.11	5.0	615	No
16	M	52	48	Bladed weapon injuries	5.1	109	0.05	5	2630	No
17	M	40	86	Hemorrhagic shock	6.4	56.8	0.05	5	3240	Yes
18	M	23	127	Road traffic accident polytrauma	80.0	5.5	0.61	5.0	4590	No
19	M	23	127	Road traffic accident polytrauma	51.3	25.0	30.0	17.4	2330	No
20	M	54	96	Falling from height	80.0	28.7	30.0	5.0	3490	No

**Table 4:** Confusion matrix using MYO values. The matrix shows the true positives (TPs), false positives (FPs), false negatives (FNs), and true negatives (TNs) for the classification of “Control” and “Experimental” groups based on MYO levels.

Observed	Predicted MYO	
	Control	Experimental
Control	13 (TP)	6 (FP)
Experimental	7 (FN)	16 (TN)

**Table 5:** Confusion matrix using TnI values. This matrix displays the TPs, FPs, FNs, and TNs for the classification of “Control” and “Experimental” groups based on TnI levels.

Observed	Predicted TnI	
	Control	Experimental
Control	13 (TP)	4 (FP)
Experimental	7 (FN)	18 (TN)

**Table 6:** Confusion matrix using MYO + TnI values. The matrix summarizes the TPs, FPs, FNs, and TNs for the combined classification of “Control” and “Experimental” groups based on both MYO and TnI levels.

Observed	Predicted MYO + TnI	
	Control	Experimental
Control	17 (TP)	5 (FP)
Experimental	3 (FN)	17 (TN)

## Data Availability

The original contributions presented in this study are included in the Supplementary Materials. Further inquiries can be directed to the corresponding author(s).

## Supplementary Materials

The following supporting information can be downloaded at: https://www.mdpi.com/article/10.3390/biomedicines13010193/s1.
